# How parents’ feeding styles, attitudes, and multifactorial aspects are associated with feeding difficulties in children

**DOI:** 10.1186/s12887-023-04369-4

**Published:** 2023-10-28

**Authors:** Evelin Czarny Hasbani, Paula Victória Félix, Patricia Kawai Sauan, Priscila Maximino, Rachel Helena Vieira Machado, Gerson Ferrari, Mauro Fisberg

**Affiliations:** 1 Sciences Applied to Pediatrics Postgraduate Program, Federal University of São Paulo, Rua Botucatu, 598, Vila Clementino, São Paulo, 04023-062 SP Brazil; 2 School of Public Health, University of São Paulo, Av. Dr. Arnaldo, 715, São Paulo, 01246-904 SP Brazil; 3 Consulting and Training, Rua José Maria Lisboa, 586, São Paulo, 01423-000 SP Brazil; 4 CENDA (Excellency Center for Nutrition and Feeding Difficulties), Instituto PENSI-Jose Luiz E. Setubal Foundation, Av Angelica 2071, CEP 01227-200, São Paulo, Brazil; 5 HCor Research Institute, Rua Abílio Soares, 250, São Paulo, São Paulo, CEP 04005909 SP Brazil; 6 Escuela de Ciencias de la Actividad Física, el Deporte y la Salud, Universidad de Santiago de Chile (USACH), Santiago, Chile; 7 Facultad de Ciencias de la Salud, Universidad Autónoma de Chile, Providencia, Chile

**Keywords:** Feeding styles, Feeding difficulties, Parental practices, Children, Meals

## Abstract

**Background:**

Parental complaints about feeding difficulties (FD) during childhood are frequent in pediatrics. Behavioral factors about children’s feeding and parental aspects are fundamental in solving these problems, but research in this area lacks information considering the joint presence of fathers and mothers. Thus, this study aimed to investigate the features of children, parents and mealtime practices related to FD reported by fathers and mothers and to identify parenting styles, mealtime actions, practices and factors associated with FD in children.

**Methods:**

323 parents (226 mothers and 97 fathers) of children aged 1 to 7 years were recruited in the emergency waiting room at Sabará Hospital Infantil, in São Paulo, Brazil, and self-completed electronic questionnaires on parenting style (Caregiver’s Feeding Styles Questionnaire), parents’ mealtime actions (Parent Mealtime Action Scale), socioeconomic information, personal and children’s health data and routine meal practices.

**Results:**

The prevalence of FD in children was 26.6%. Indulgent parenting style was the most frequent (44.2%), followed by authoritarian (25.1%), authoritative (23.8%), and uninvolved (6.9%) styles. Most parents (75.8%) reported presence during meals, and 83.6% used distractions. Regression analyses after adjustments showed, as factors associated with FD, female children (OR: 2.06; 95%CI: 1.19–3.58), parents’ FD history (OR: 3.16; 95%CI: 1.77–5.64), and greater frequency of parents’ behavior of offering many food options (OR: 2.69; 95%CI: 1.18–6.14). Parents with indulgent styles had decreased chances of reporting FD in their children (OR: 0.13; 95%CI: 0.06–0.27). Furthermore, the practice of children sharing the family menu (OR: 0.43; 95%CI: 0.18–0.99) and higher frequency of parents’ behavior of setting snack limits (OR: 0.44; 95%CI: 0.23–0.85) were inversely associated with FD.

**Conclusions:**

This study reinforces the multifactorial aspects involved in the feeding difficulties context. It points out the importance of expanding knowledge of the individual role of fathers and mothers to compose a scenario that can guide future studies and interventions.

**Trial registration:**

CAAE #99221318.1.0000.5567 with registration number 2,961,598.

## Background

Feeding problems are common in childhood, a period considered critical for physical growth, neural development, and to shaping eating behaviors and habits that will continue into adulthood [1–3]. It is estimated that between 20% and 60% parents are concerned with their children’s feeding, having transient or persistent complaints that can compromise the family relationship [4]. The most common problems are related to behavioral aspects such as food selectivity, quantity variation or organic issues and are called feeding difficulties (FD) [4, 5].

Considering fathers and mothers responsible for the environment and socialization of children in the context of food [6], evidence supports that their characteristics [7], the way they react to their children’s problems related to feeding and aspects of the food environment can influence positively or harm habits and health of children [7, 8].

Parenting style is a constellation of parental attitudes and beliefs toward childrearing, creating an emotional climate through which parental practices are expressed [9, 10], including the quality of parent–child interactions. Parenting style has two independent dimensions: (1) Demandingness/control, defined as claims that parents make on children to become integrated into society by behavior regulation, direction confrontation, and maturity demand (behavioral control) and supervision of the child’s activities [10]; and (2) responsiveness/nurturance, defined as the extent to which parents foster individuality and self-assertion by being attuned, supportive, and acquiescent to children’s requests including autonomy support and reasoned communication [10]. Crossing the first two dimensions yields four categories of parenting style: (1) authoritative (high demandingness, high responsiveness) characterized by parental involvement, nurturance, and expectations with monitoring; (2) authoritarian (high demandingness, low responsiveness) characterized by restrictive, punitive, and power-assertive behaviors; (3) indulgent (low demandingness and high responsiveness) characterized by warmth and acceptance in conjunction with a lack of monitoring of child behavior; and (4) uninvolved (low demanding, low responsive), characterized by little control, nurturance, or involvement with the child [11, 12]. Otherwise, parental feeding practices are strategies and behaviors adopted by parents with specific and direct goals in child’s feeding scope involving pressure to eat, use of rewards, encouragement, rules in eating, among others discussed in the literature [12].

Studies demonstrate the importance of father inclusion in investigations related to parenting, due to its influence on eating habits and the greater current participation in children’s lives and mealtime routine. However, the literature in the field of children’s nutrition was mostly built with data from mothers, lacking information from the underrepresented fathers [13, 14].

Thus, understanding attitudes, behaviors and dynamics in feeding, considering fathers and mothers is relevant to understand the factors that contribute to FD in children and to help families develop a healthy relationship and better eating behaviors [15–18]. Therefore, in order to contribute to the gap showed in the literature, this study aimed to investigate the features of children, parents and mealtime practices related to FD reported by fathers and mothers and to identify parenting styles, mealtime actions, practices and factors associated with FD in children.

## Methods

### Study design and sample

This is a quantitative observational cross-sectional study prepared on the Google Forms® online platform. From October 2018 to November 2019 fathers or mothers of different one to seven years old children rated with less severe symptoms or illnesses according to the screening sector of the Emergency Room from Sabará Hospital Infantil in the city of São Paulo (Brazil) were invited to take part in the study. Participation was voluntary to receive a link to a lecture on the work theme, “Feeding Difficulties in Childhood” as a thank you to the participants.

The sampling size was calculated with a confidence level of 95%, a maximum error of 3.49%, and 80% sampling power. Furthermore, the calculations of the minimum sample sizes required per strata (i.e., one third of fathers and two thirds of mother) were performed using the GPower® 3.0 program resulting in a required sample size of 287 children aged 1–7 years of age.

This study was conducted according to the guidelines laid down in the Declaration of Helsinki and all procedures involving human subjects/patients was approved by the ethical review board. Ethical approval was granted by the Ethics Committee of Instituto PENSI do Sabará Hospital Infantil and Universidade Federal de São Paulo (UNIFESP) under CAAE # 99221318.1.0000.5567 with registration number 2.961.598. After clarification about the research and guarantee of data confidentiality, those who agreed to participate signed the informed consent form and proceeded to self-complete the electronic survey questionnaire on tablets or cell phones, lasting approximately 20 min.

A semi-structured questionnaire was applied in order to gather the child’s identification data (name, gender, date of birth), identification of parents (name, gender, age, relationship with the child, email or WhatsApp®), and socioeconomic data (self-reported race, parents’ education level, monthly family income, marital status, working hours, number of children of parents). The questionnaire also has questions about the conditions of the child’s birth (birth order, and prematurity: considered as < 37 weeks [19]), medical history (presence of diseases) and parents’ previous diagnosis of depression, feeding disorders and history of FD were investigated.

### Independent variables

#### Anthropometric measurements

The children’s weight and height were measured in a room used for screening in the emergency. The children were accompanied by their parents, and the protocol followed the guidelines of the World Health Organization (WHO) [20], adopted by the Food and Nutritional Surveillance System [21]. Subsequently, the nutritional status was determined by the body mass index (BMI) for age (BMIZ), and length/height for age (HAZ) and classified by z-score using the WHO Anthro and WHO Anthro Plus programs. The weight and height of the parents were reported by them, and the nutritional status was defined by the BMI according to the parameters of the WHO [22]. The principal investigator was responsible for the anthropometric measurements. During four months, two interns, students of nutrition, supervised by the principal investigator helped with the data collection and each one of them stayed for two months.

#### Feeding styles

The Caregiver’s Feeding Styles Questionnaire (CFSQ) [23], validated for the Brazilian population, was used to rate four parenting styles: authoritative, authoritarian, indulgent, and uninvolved [24]. Feeding styles refer to the overall attitude of parents that results in general patterns of behaviors that parents apply when feeding their children based on the dimensions of responsiveness and demandingness  [23]. This instrument consists of 19 questions organized on a five-point Likert scale (never = 1, rarely = 2, sometimes = 3, almost always = 4, and always = 5) having the frequency of certain parental guidelines in feeding to access parenting styles through two dimensions: demand and responsiveness [24]. The score calculation (demand = 2.8 and responsiveness = 1.16) is performed for the classification and definition of styles (authoritative = high demand/high responsiveness; authoritarian = high demand/low responsiveness; indulgent = low demand/high responsiveness; uninvolved = low demand/low responsiveness). The scores were defined after the analyses of five separate empirical studies. Two constructs encompass these dimensions “parent-centered feeding strategies” (with 12 items) and “child-centered feeding strategies” (with 7 items). The assessment of “demandingness” is given by the average score of the 19 items (“parent-centered feeding strategies” and “child-centered feeding strategies”), and the assessment of “responsiveness” is calculated by the ratio of the mean score of the seven items (“child-centered feeding strategies” 3 + 4 + 6 + 8 + 9 + 15 + 17), subdivided by the total mean score of the 19 items (“parent-centered feeding strategies” and “child-centered feeding strategies”). Demandingness represents how much a parent encourages her/his child to eat, while responsiveness represents how parents encourage their children to eat [11].

#### Parent mealtime action scale (PMAS)

Fathers or mothers answered a 31 items-scale to assess the frequency (never = 1, sometimes = 2, always = 3) of behaviors. They presented behaviors divided into nine domains: daily fruits and vegetables availability (FVA), snack modeling (SM), use of rewards (UR), many food choices (MFC), fat reduction (FR), special meal (SpM), snack limits (SLs), positive persuasion (PP). The result was obtained by calculating the arithmetic mean of each behavior according to the original version of the scale, indicating that the higher the value obtained in the behavior domain, the more frequently the parents practiced that behavior [25, 26]. The Parent Mealtime Action Scale has been validated to evaluate the influence of parent mealtime actions on their children´s food intake in Brazil with correlation coefficients ranging from 0.38 to 0.80 

 [26].

#### Mealtime practices

To assess other practices in the family food environment that could be related to FD, a research questionnaire was developed based on the literature [27–29] and clinical practice to gather data on the place of meals, caregiver presence, use of distractions, the child’s sharing of the family menu, and frequency of meals shared with the child. The frequency of shared parent’s meals was categorized as ≤ 14 and > 14 meals per week (presence of the parents at the meals ≥ 2 times per day) due to the lack of definition of a cutoff point showed in a meta-analysis [30] and systematic review [31].

### Dependent variable

Based on previous research [32, 33], the presence of FD was addressed according to the complaint or perception of parents about the problem through the question: “Does your child give you a hard time to eat? Does it to the point where you worry, disturb the family’s routine and you do think about asking for help?”.

### Statistical analysis

Data analysis and processing were performed using Stata® software (version 14.0, 2011, Stata Corp LP). Descriptive statistics, including mean, standard deviation, absolute, and proportions were calculated for the independent variables by FD (no vs. yes). To compare two categorical variables, the Chi-square test or Fisher’s exact test was used.

The association between the independent variables with FD (0 = without FD vs. 1 = with FD) was tested by binary logistic regression models (odds ratio: OR; 95% confidence interval: 95% CI) crude and adjusted were performed. In the first instance, sociodemographic variables and parent styles were entered in univariate models. The variables that were significant at p < 0.20 were included in mutually adjusted models for (i.e. child’s gender, guardian’s gender, child’s age, history of diseases and parental style classification). In the second instance, we ran univariate and multivariate logistic to evaluate the association between parents’ feeding behaviors with FD. The models were adjusted for child’s gender, guardian’s gender, child’s age, history of diseases and parental style classification. The reference category for regression was the child´s FD. A significance level of less than 5% was considered.

## Results

The children’s mean age was 3.5 ± 1.6 years old; 51.1% were male, full-term born (85.9%), eutrophic (72.1%), and with adequate stature for their age (96.8%). Data related to the children’s characteristics are shown in Table 1. The prevalence of FD was 26.6% (n = 86). There was a significant difference between gender and FD (p = 0.024). The body mass (BMIZ) and height-for age (HAZ) indexes, child age, and history of previous diseases did not show differences between the groups with and without FD reported by the parents, as well as variables prematurity (p = 0.798) and child-birth order (p = 0.465) (Table 1).

**Table 1:** General characteristic of children according to feeding difficulties.

Variables	Total population (n = 323)			FD (child)				p*
				No (n = 237)		Yes (n = 86)		
	n		%	n	%	n	%	
**Gender**								
Male	165		51.08	130	54.85	35	40.70	0.024
Female	158		48.92	107	45.15	51	59.30	
**Age in years**								
< 3	109		33.75	75	31.65	34	39.53	0.415
≥ 3 to < 5 years	131		40.56	99	41.77	32	37.21	
≥ 5 years	83		25.70	63	26.58	20	23.26	
**BMIZ**								
Eutrophic	204		72.08	147	71.71	57	73.08	0.818
Overweight	79		27.92	58	28.29	21	26.92	
**HAZ**								
Very low/low	9		3.18	5	2.44	4	5.13	0.266
Appropriate	274		96.82	200	97.56	74	94.87	
**History of diseases**								
Respiratory diseases (n = 323)	143		44.27	103	43.46	40	46.51	0.626
Food allergy (n = 210)	25		11.90	20	12.74	5	9.43	0.521
Gastrointestinal disease (n = 209)	22		10.53	14	8.97	8	15.09	0.210
Endocrine disease (n = 209)	7		3.35	4	2.56	3	5.66	0.373
Kidney disease (n = 209)	3		1.44	1	0.64	2	3.77	0.159
Neurological disease (n = 209)	4		1.91	1	0.64	3	5.66	0.051
**Prematurity**								
Yes	43	14.14		31	13.84	12	15.00	0.798
No	261	85.86		193	86.16	68	85.00	
**Birth order**								
1	210	66.25		152	65.24	58	69.05	0.465
2	73	23.03		53	22.75	20	23.81	
3	34	10.73		28	12.02	6	7.14	

Note: * Significance seen by the chi-square or Fisher test; BMI/A = body mass index for age; % = percentage; n = sample; significance level p < 0.05. FD = feeding difficulties; HAZ = height for age

Data on general characteristics of fathers (n = 97) and mothers (n = 226) are shown in Table 2. Most individuals declared themselves white (80.0%), with a high level of education (77.7%), family income above 10 minimum wages (46.1%) and 44 h per week or more of work (57.8%). There was a significant effect in relation to the outcome history of FD of those parents (p < 0.001), previous diagnosis of depression (p = 0.005) and parenting styles (p < 0.001) (Table 2).

**Table 2:** General characteristic of parents according to feeding difficulties.

Parents variables	Total population (n = 323)		FD (child)				p*
			No (n = 237)		Yes (n = 86)		
	n	%	n	%	n	%	
**Gender**							
Female	226	69.97	161	67.93	65	75.58	0.185
Male	97	30.03	76	32.07	21	24.42	
**Self-reported skin color**							
White	248	80.00	185	80.79	63	77.78	0.561
Not white	62	20.00	44	19.21	18	22.22	
**Education level**							
Up to high school	72	22.29	54	22.78	18	20.93	0.905
Higher education	159	49.23	115	48.52	44	51.16	
Above higher education	92	28.48	68	28.69	24	27.91	
**Working regime**							
Not engaged in paid activity	64	22.54	46	22.33	18	23.08	0.828
Part-time (< 44 h per week)	56	19.72	39	18.93	17	21.79	
Full-time (≥ 44 h per week)	164	57.75	121	58.74	43	55.13	
**Income category**							
≤ 3 MW	28	8.78	20	8.55	8	9.41	0.182
> 3 to 10 MW	144	45.14	99	42.31	45	52.94	
> 10 MW	147	46.08	115	49.15	32	37.65	
**Marital status**							
With partner	284	89.31	208	88.89	76	90.48	0.686
With no partner	34	10.69	26	11.11	8	9.52	
**Number of children**							
1	164	51.74	118	50.64	46	54.76	0.294
2	120	37.85	87	37.34	33	39.29	
≥ 3	33	10.41	28	12.02	5	5.95	
**Nutritional status**							
Low weight/eutrophic	130	42.07	96	41.74	34	43.04	0.933
Overweight	107	34.63	81	35.22	26	32.91	
Obesity	72	23.30	53	23.04	19	24.05	
**Prior health history**							
Diag. of depression	46	15.23	26	11.71	20	25.00	0.005
Diag. of eating disorder	10	3.31	6	2.69	4	5.06	0.295
FD	115	36.28	67	28.76	48	57.14	< 0.001
**Parental style**							
Authoritative	76	23.82	42	18.03	34	39.53	< 0.001
Authoritarian	80	25.08	46	19.74	34	39.53	
Indulgent	141	44.20	127	54.51	14	16.28	
Uninvolved	22	6.90	18	7.73	4	4.65	

Note: Minimum wage = R$ 1,045.00; Significance seen by the chi-square test or Fisher’s test; n = sample; significance level p < 0.05. FD = feeding difficulties

Data related to routine mealtime practices are shown in Table 3. The majority (81.73%) of fathers and mothers reported having meals in an appropriate place (at the table) and using distraction (television, tablet or cell phone) during meals (83.59%) with some frequency.

**Table 3:** Descriptive mealtime practices according to feeding difficulties.

Variables	Total Population (n = 323)		FD (child)				p*
			No (n = 237)		Yes (n = 86)		
	n	%	n	%	n	%	
**Place of meals**							
Table	264	81.73	198	83.54	66	76.74	0.162
Others	59	18.20	39	16.46	20	23.26	
**Use of media**							
Never	53	16.41	41	17.30	12	13.95	0.473
Sometimes/always	270	83.59	196	82.70	74	86.05	
**Child sharing family menu**							
No	34	11.11	20	8.85	14	17.50	0.034
Yes	272	88.89	206	91.15	66	82.50	
**Person in charge of preparing meals**							
Mother	206	67.32	147	65.04	59	73.75	0.270
Father	22	7.19	17	7.52	5	6.25	
Grandparents	51	16.67	38	16.81	13	16.25	
Employees/others	27	8.82	24	10.62	3	3.75	
**Parent presence at meals**							
Yes	244	75.78	180	75.95	64	75.29	0.904
No	78	24.22	57	24.05	21	24.71	
**Shared weekly meals**							
≤ 14	170	52.63	125	52.74	45	52.33	0.947
> 14	153	47.37	112	47.26	41	47.67	

Note: * Significance seen by Chi-square test or Fisher’s test; n = sample; significance level p < 0.05. Weekly meals: breakfast + lunch + dinner. FD = feeding difficulties

Fathers and/or mothers were present during meals (75.78%) and 47.37% reported sharing > 14 main meals a week with the child (breakfast or lunch or dinner). The child sharing the family menu (eating the same meal prepared for the family) showed a significant result in relation to FD (p = 0.034) (Table 3).

In the logistic regression model showed in Table 4, after adjustments, factors associated with FD were presented as female children (OR: 2.06; 95%CI: 1.19–3.58), parents with an FD history (OR: 3.16; 95%CI: 1.77 – 5.64). Parents classified as indulgent showed reduced chances of perceiving FD in their children (OR: 0.13; 95%CI: 0.06 – 0.27) in relation to authoritative styles. The sharing of the family menu with the child (eating the same meal as the family) was inversely associated with FD (OR: 0.43; 95%CI: 0.18 – 0.99).

**Table 4:** Logistic regression models sociodemographic variables and parent styles for feeding difficulties.

Predictors	n ^a^	OR	95%CI	p
Gender of parents (male)	319	0.57	(0.31–1.05)	0.073
Gender of child (female)	319	2.06	(1.19–3.58)	0.010
Child age (years old)	319	0.89	(0.75–1.06)	0.204
Parent style (authoritative)	319			
Authoritarian		1.00	(0.52–1.92)	0.994
Indulgent		0.13	(0.06–0.27)	< 0.001
Uninvolved		0.36	(0.11–1.21)	0.098
Parents’ depression (yes)	298	2.05	(0.98–4.28)	0.056
Parents’ FD (yes)	313	3.16	(1.77–5.64)	< 0.001
Child share family menu (yes)	302	0.43	(0.18–0.99)	0.049

Note: Mutually adjusted model for child’s gender, guardian’s gender, child’s age, history of diseases and parental style classification

Category of reference: = presence of FD in children reported by fathers and mothers; FD = feeding difficulties; OR = odds ratio; 95%CI = 95% confidence interval; ^**a**^: sample size for each regression model; significance level p < 0.05

After adjusting the multivariate logistic regression model in Table 5, it was seen that the increase of one unit in the many food choices behavior domain was positively associated with FD (OR: 2.69; 95%CI: 1.18 – 6.14) while higher values in the domain snack limits indicated decreased chances for FD (OR: 0.44; 95%CI: 0.23 – 0.85) (Table 5).

**Table 5:** Logistic regression models of parents’ feeding behaviors associated for feeding difficulties.

PMAS Questionnaire	Crude				Adjusted*			
	n ^a^	OR	95%CI	p	n	OR	95%CI	p
Daily availability of fruits and vegetables	313	0.54	(0.19–0.99)	0.048	311	0.52	(0.26–1.05)	0.068
Snack modeling	315	1.63	(0.88–3.01)	0.118	312	1.19	(0.61–2.33)	0.609
Use of reward	315	1.99	(1.05–3.79)	0.035	312	0.67	(0.31–1.52)	0.353
Many food options	314	3.72	(1.77–7.80)	0.001	311	2.69	(1.18–6.14)	0.019
Fat reduction	316	0.69	(0.39–1.22)	0.205	313	0.80	(0.43–1.49)	0.483
Special meals	313	1.04	(0.39–2.79)	0.941	311	0.63	(0.19–2.03)	0.442
Snack limits	315	0.57	(0.33–1.01)	0.053	312	0.44	(0.23–0.85)	0.014
Positive persuasion	314	2.61	(1.38–4.96)	0.003	312	1.56	(0.75–3.24)	0.230
Insistence on eating	315	1.46	(0.89–2.38)	0.128	312	1.06	(0.60–1.88)	0.840

Note: *Model adjusted for child’s gender, guardian’s gender, child’s age, history of diseases and parental style classification; PMAS = Parent Mealtime Action Scale; OR = odds ratio; 95%CI = 95% confidence interval; ^a^ sample size for each regression model; significance level p < 0.05

## Discussion

Regarding the study’s main findings, the FD was positively associated with female children, FD history of fathers and mothers, and greater frequency of parents’ behavior of offering many food options to the child. Indulgent parental style, the practice of child sharing the family menu, and higher frequency of parents’ behavior of establishing snack limits were related to lower chances of FD.

There is no current consensus on terms to define or diagnose pediatric feeding problems, nor is there a valid and reliable method of assessment or evaluation. Reviews of the literature on pediatric feeding problems and disorders repeatedly reference the lack of a shared conceptualization of feeding problems [34]. Some limitations of studies should also be taken into account. Some studies simply use a parental description of picky eating [35, 36]. Despite variation on the definition of problematic eating behaviours, these include food refusal (of certain types of foods), food fussiness or pickiness (refusal of new and familiar foods, accepting only a narrow range of foods), refusal of new foods (neophobia), grumpiness during mealtime and inadequate self-feeding skills [36–39]. It´s recognized that term FD is a useful umbrella term that simply suggests there is a feeding problem of some sort. In essence, if the mother says there’s a problem, there is a problem [40]. There are many areas of research that still need to be addressed including health-related outcomes [41].

The prevalence of feeding problems may vary between 7% and 65% [38, 39]. According to the parents’ reports in our study, the prevalence of FD was 26.6%. This results was similar to the findings by Benjasuwantep et al., who classified 26.9% of feeding problems in 1–4 years old Thai children treated at a medical center [42] and the Brazilian study by Maranhão et al. in which the FD reported by mothers was 25.1% in a population of 2–6 years old children [43].

Regarding the child gender, it is described in the literature that parents tend to express greater concern about their daughters’ weight and nutrition than their sons’ [44, 45]. In this sense, social standards related to body image and weight of women culturally highlighted in the West and reinforced by social media [46, 47] can influence the differences in parents’ behaviors related to feeding girls against boys [48, 49] and in the greater perception of difficulties in the girls [49]. Although results relating to gender and children’s FD in the literature are contradictory [50], research suggests parental feeding practices can contribute to the difference between the genders [51] and impact the behavior of preschool and school-age boys and girls in different ways [51, 52]. In this research, the results were consistent with the work by Cao et al., who found higher FD (food fussiness) scores in girls [53].

The finding of a lower chance of FD inference by parents with an Indulgent parental style suggested a link to the characteristics of this style, which expresses less expectation, monitoring, and structure about food and offers greater freedom of food choice, possibly tending not to notice problems [51, 52]. These results are noteworthy because, according to the literature, children of Indulgent parents have a lower ability to self-regulate their diet and have higher BMI values than other parental styles [44, 54, 55].

It is widely appreciated that fathers, mothers, and caregivers are role models in children’s nutrition and are primarily responsible for food exposure [25, 26], therefore, their experiences with FD may be relevant to FD conditions in their children [56, 57]. As expected, in the current study, fathers and mothers with an FD history were likelier to report the problem in their children. Although the information on the consumption of caregivers and children has not been collected, allowing specific comparisons, the parent’s experience of FD could lead to the exclusion of their non-tolerated foods from the food environment of the child, generally healthy food. In addition, the child may miss the opportunity to learn through the parental model, which makes it challenging to expand the food repertoire [26, 58] even if the child is exposed to the foods desired by the parents. Still, in this context, given that those parents have shown remission of the FD developed in childhood, the experience could lead to more significant concern and perception of problems in their children [56], but these aspects should be further investigated.

In this research, the mealtime practice of children sharing the same family menu was inversely associated with FD, which was favorable to a child’s food acceptance, in accordance with the literature [16, 59]. Similarly, Powell et al., [59] in a study with 2–4 years old American children, found less food refusal and easier feeding of children who did not use distractions and who had the practice of consuming the same meal as their mothers. In the present study, attention is drawn to the use of distractions during meals by most children, even though it was unrelated to FD. Still, considering that this is a coercive practice that impairs the perception of hunger and satiety at mealtime, it should not be reinforced at any time [60].

Anxiety and concern about the children being considered challenging to eat can model unresponsive practices [61]. Similarly, in this study, the tradition of offering many food options to the child was positively associated with FD. According to the literature, the preparation of favorite dishes and avoiding conflicts at meals are strategies that, in addition to affecting the structure of meals [61], can reinforce the cycle of refusal and lead children to more restricted diets and lower consumption of fruits and vegetables [25, 32, 62]. Given these findings, it is recommended that the options be guided within a limit of the preparations offered [63]. Still, a child’s autonomy and involvement in food choices should always be encouraged [16].

Similarly, regarding the behavior of setting snack limits, a study with 5185 3–5 years old children identified that the parents’ permissive profile concerning their children’s demands for snacks had a negative influence on the quality of snacks consumed [64]. It is recognized that selective children tend to have a strong preference for sweets or treats (snacks), which may be further reinforced by the propensity of their parents to offer this type of snack during or between meals [65]. Although a higher frequency of indulgent parents (44.8%) has been observed in this study, the practice of establishing snack limits proved beneficial when related to fewer chances of reports of FD. It’s worth noting that there are different ways of instituting control in children’s feeding context. From a perspective of restriction, generally characteristic of authoritarian parents, it can promote a negative impact, such as worsening the child´s FD, changing nutritional status, affecting signs of hunger and satiety, and stress and concern for the caregiver [4, 16, 49, 52].

Thus, the strategies promoting and supporting healthy practices among children generally involve responsive parenting, feeding, and a healthy food environment that involves sharing meals, menus, established rules, and pleasure during meals [29, 66].

The current study adds to the literature by jointly accessing styles, feeding practices, and relevant factors for children’s nutrition using a sample that considers fathers and mothers. International works have been pointing the relevance of including father in research about children´s feeding subjects, but it´s observed an absence of Brazilian studies related to parental style and feeding practices [13, 14, 67]; however, the limitations must be considered. This work is cross-sectional, which limits the assertion of causality between the findings or whether those responsible were reacting to the children or vice-versa. It includes a convenience sample of fathers and mothers with a high socioeconomic profile, primarily white, residents of São Paulo, and children with a defined age group, thus not allowing the generalization of data. However, the instruments used were validated with similar populations. Data on children’s consumption were not collected for diet analysis and FD classification for comparison with the parents’ reports. Still, a more straightforward way of questioning, compatible with the shape of other studies, was used to assess the perception of fathers and mothers about children’s feeding problems. The research was self-responded in an emergency room, a place of greater tension for those responsible. Still, care was taken to prioritize the order of care established by the hospital and offer play activities for the children. At the same time, their parents answered the research, as well as the choice of electronic format to facilitate participation. The strengths of this study include fathers and mother’s information, without gender distinction, suggesting a global look. These results are relevant because include parents in the feeding scenario, meeting the gap presented by the literature on the generalization of outcomes in children’s feeding based on research carried out only with mothers. In addition, it highlighted permissive eating styles among fathers and mothers and important factors associated with the perception of eating difficulties.

## Conclusion

This study showed that the female gender, parents’ FD history, and greater frequency of parents’ behavior of offering many food options were positively associated with children’s FD.

Indulgent parental style, the practice of child’s sharing of family menu during meals, and higher frequency of parents’ behavior snack limits were inversely related to children’s FD.

This work reinforces the multifactorial aspects involved in children’s FD. In addition, the results point to the importance of expanding knowledge of the individual role of fathers and mothers, as well as their dynamics as couples and other caregivers present in food, to compose scenarios closer to reality to guide future studies and intervention proposals to promote an adequate food environment for the child.

## Data Availability

The dataset used and analysed during the current study are available from the corresponding author on reasonable request.
